# Opioid-related harms and experiences of care among people in justice settings in New South Wales, Australia: evidence from the National Ambulance Surveillance System

**DOI:** 10.1186/s12954-025-01154-7

**Published:** 2025-01-15

**Authors:** Naomi Beard, Michael McGrath, Harry M. X. Lai, James Wilson, Anthony Hew, Amaya Muñoz Labiano, Dan I. Lubman, Rowan P. Ogeil

**Affiliations:** 1 Turning Point, Eastern Health, Richmond, VIC Australia; 2 Faculty of Medicine and Health, University of New South Wales, Sydney, NSW, Australia; 3 Eastern Health Clinical School and Monash Addiction Research Centre, Monash University, Box Hill, VIC, Australia; 4 Health Data Intelligence, New South Wales Ambulance, State Operations Centre, Sydney, NSW Australia; 5 Faculty of Medicine and Health, University of Sydney, Sydney, NSW, Australia; 6 Barwon Health, Geelong, VIC, Australia

**Keywords:** Overdose, Paramedicine, Opioid-use disorder, Harm reduction, Opioid withdrawal, Blood-borne viruses, Justice health

## Abstract

**Background:**

People in justice settings experience higher rates of psychiatric morbidity, including alcohol and drug use disorders, compared with the general population. However, our understanding of opioid-related harms in justice settings is limited. This study used ambulance data to examine opioid-related harms and experiences of care in New South Wales (NSW), Australia, during periods of incarceration or detention.

**Methods:**

This mixed-methods study used data from the National Ambulance Surveillance System (NASS) for patients aged 18 and older with an opioid-related ambulance attendance between December 2020 and April 2023. People in justice settings were identified using ambulance billing codes and manual review of paramedic case notes. Descriptive statistics described the patterns and modalities of opioid-related harms in justice settings, and a qualitative thematic analysis of paramedic case notes was used to contextualise findings. Results Over the study period, 328 opioid-related ambulance attendances for people in justice settings were identified (51% heroin; 41% opioid agonist therapy (OAT) medication). Symptoms of opioid withdrawal were noted in 35% of attendances, most commonly for heroin (51%) and withdrawal from OAT medications (48%). Three interconnected themes were identified using qualitative analysis: trust and mistrust in justice settings, systemic barriers to providing OAT, and other harm reduction strategies, and experiences of withdrawal in justice settings.

**Conclusion:**

Our study demonstrated the utility of ambulance data in identifying opioid-related harms for people in justice settings in NSW. Qualitative findings highlight current barriers to effective opioid care in justice settings and identify opportunities for intervention, including targeted harm reduction programs, as well as policies that promote continuity of care particularly during transitions in and out of justice settings.

## Introduction

People in justice settings experience higher rates of psychiatric morbidity, including depression, anxiety, suicidal behaviours, and alcohol and drug (AOD) harms compared with the general population [1– 3]. Furthermore, a consistently higher prevalence of blood-borne viruses (BBV) has been observed among people in justice settings, particularly in those who inject drugs [4, 5]. People with a history of imprisonment experienced elevated all-cause mortality compared with the general population, with the greatest disparities seen in deaths related to suicide, poisonings and drug-related causes [6, 7]. People who use drugs constitute a wide range of people in prison, with estimates varying on the drugs assessed, the location, and the methodology employed. For example, studies across multiple European countries have found that between 15 and 80% of people entering prison report lifetime use of any illicit drug, with 5–40% reporting heroin use [8, 9]. In a 2020 survey of people in prison in New South Wales (NSW), Australia, more than half (54%) of respondents reported drug use in the four weeks prior to entry to prison, with 19% reporting intravenous drug use [10]. In terms of opioid use, a NSW 2015 prison survey found that one-third of respondents reported lifetime extra-medical use of a prescribed analgesic (33%), methadone or buprenorphine (35%), or heroin (41%), highlighting the widespread use of opioids among people in justice settings [11].

Evidence-based interventions for reducing harms related to opioid dependence in the community include needle and syringe programs, peer-led education, opioid agonist therapy (OAT), naloxone provision and BBV screening and treatment [12]. However, access to these programs in justice settings is limited [13, 14]. The geographic, economic, political and sociocultural factors affecting the availability and accessibility of these interventions in justice settings vary globally [13, 14], but issues associated with stigma, concerns related to diversion, and insufficient training or confidence among police and correctional staff to respond effectively during an overdose are common across jurisdictions [15– 17].

NSW has the largest number of people receiving OAT in correctional facilities in Australia, with more than 3,600 people in prison receiving treatment in 2023 [18]. Upon entry to prison in NSW, people who have accessed OAT programs in the community experience delays in treatment and difficulties navigating prison OAT systems, while those not previously on an OAT program face additional hurdles [19]. Furthermore, delays and interruptions to accessing their prescribed medications, including OAT and other prescription medications (e.g. antipsychotics, antidepressants, anticonvulsants and direct acting antivirals), highlights challenges with continuity of care in justice-settings [13, 20].

To date, the literature on opioids in justice settings has primarily focussed on harm reduction policies implemented at prison entry and release, with few studies examining harms during periods of incarceration [21, 22]. This limits our understanding of the prevalence and context surrounding harms experienced in these settings, particularly when external paramedic attendance or transport to hospital is required. Ambulance services are essential emergency healthcare responders, frequently attending to incidents of AOD-related harms in the community [23]. In NSW, people in prison can seek and receive medical care through the prison health clinic, however in more severe medical emergencies, including instances of opioid overdose, ambulances are an important provider of pre-hospital care. Ambulances are provided by NSW Ambulance, a single state-wide provider of emergency and pre-hospital healthcare. For people who are in prison or in police custody in NSW, ambulance care is provided at no cost to the individual and these cases are assigned a unique administrative billing code. Ambulance attendance data have demonstrated utility for assessing drug-related harms in the community [24, 25], and may overcome methodological shortcomings associated with survey-based or interview methods which include non-disclosure for fear of criminal sanction [15, 26].

To gain greater contextualisation of opioid-related harms in justice settings, the present study utilised a novel surveillance system that captures alcohol and drug-related attendances from NSW, Australia and aimed to: *i)* describe the patterns of harms related to opioids for people in justice settings when an ambulance attendance was required, and *ii*) contextualise the experiences of people in justice settings with opioid-related harms and the implications those harms have on health service delivery and systems.

## Methods

### Data source

NSW data were obtained from the National Ambulance Surveillance System (NASS), an internationally unique monitoring system for acute harms involving AOD, mental health, and suicide and self-harm [23, 27]. A filtered dataset comprised of ambulance attendance records involving AOD, mental health or self-harm are sent to the NASS by jurisdictional ambulance services. Attendances are given a unique patient identifier, which includes demographic, geographic and clinical details. Data are imported to a custom-built coding database, where patient records are examined by trained research assistants following validated guidelines [23, 27]. Opioid-related attendances are coded when cases meet the following criterion: ‘Is it reasonable to attribute the immediate or recent opioid use, or withdrawal, as a contributing reason for the ambulance attendance?’, noting that all attendances related to prescription opioids or OAT are considered extra-medical or over/inappropriate use of the substance where consumption was determined. If a patient described consuming a prescription opioid medication as prescribed, this would not be captured in the NASS. This study follows the ‘Reporting of Studies Conducted using Observational Routinely-collected Health Data’ (RECORD) guidelines. The NASS and methods were approved by the Eastern Health Research Ethics Committee.

### Inclusion/exclusion criteria

All opioid-related ambulance attendances in NSW between December 2020 and April 2023 for patients aged 18 and older were extracted from the NASS. For this study, an opioid-related harm includes attendances involving heroin, prescription opioid analgesics including codeine, dextropropoxyphene, fentanyl, hydromorphone, morphine, oxycodone, pethidine, tramadol, other unspecified opioid analgesics, and opioid agonist therapies (methadone, buprenorphine, and buprenorphine naloxone). In Australia, it is the responsibility of the jurisdictional justice service to provide appropriate medical care to people in police custody and correctional facilities. Attendances for people in justice settings were identified using the patient billing codes ‘*correctional services’* or ‘*police custody*’, which are assigned by NSW Ambulance, followed by a manual review of the case notes recorded by paramedics during the attendance. These billing codes capture a range of people in justice settings, including people in prison, remanded in police custody, or being transferred between sentencing and prison. Each attendance in this study represents one patient. In the NASS, police co-attended ambulance attendances can represent events that are distinctly different from events where the patient is in police custody. For example, the police may have been on scene for purpose of mental health assessments, for welfare checks or cases occurring in a police station where the patient was not in police custody. During the qualitative review of the case notes, cases where police co-attended the scene with paramedics but did not detain or arrest the patient, or where police detained the patient for the purposes of an involuntary treatment as a mental health patient (i.e., sectioning) were excluded. Cases were excluded from analysis where gender identity was missing or inadequately defined to protect patient anonymity.

### Analysis

The study employed a mixed methods approach, incorporating quantitative analysis of the NASS surveillance system and qualitative thematic analysis of paramedic case notes to contextualise opioid-related harms in the justice system [28]. The aim of the quantitative analysis was to describe the patterns of harms related to opioids for people in justice settings when an ambulance attendance was required. Descriptive statistics were used to examine patient demographics (gender and age), whether a patient was transported to hospital, and contextual characteristics (e.g., nature of attendance and opioid involved). Student’s t-test was used to examine differences in continuous variables, and Chi-squared tests (X^2^) used to examine differences in proportions between people in prison and people in police custody. Contextual characteristics are not mutually exclusive in this study, and a single attendance could involve more than one drug type for example. To protect patient anonymity, all numbers less than five are suppressed. All quantitative analyses were conducted using StataMP v15.

Two authors independently examined the qualitative data contained in the paramedic case notes of all attendances meeting our inclusion criteria. The case notes contain important contextual, behavioural, and clinical observations recorded during an ambulance attendance based on paramedic observation and expertise, and information provided by patients, bystanders, and others on scene. Case notes are recorded at the conclusion of the ambulance attendance and describe the nature of the attendance using a combination of short-hand script, clinical terminology, and professional abbreviations. Following data familiarisation and discussion, two authors independently coded the data using both deductive and inductive approaches. Themes and subthemes were generated through iterative coding based on preconceived clinical and policy priorities determined from the literature, and the preliminary quantitative analysis (for example drug use modalities), while additional codes were developed directly from the data (such as drug withdrawal experiences and processes of care). An initial coding framework of themes and subthemes was developed independently by both authors before both frameworks were merged and iteratively refined via discussion. All qualitative analyses were conducted on original paramedic case notes and reviewed in their raw form in Microsoft Excel. In the results, notes have been presented in longhand with the translation of paramedic abbreviations, (e.g., O/A: “On arrival”, Pt: “Patient”) and in a deidentified format, removing patient age and the location of attendance (e.g., name of the city, prison, or hospital).

## Results

Between December 2020 and April 2023, there were 328 opioid-related attendances for patients in justice settings, 78 occurring in prisons (P) and 250 in police custody (PC) (Table 1). These attendances were primarily for male patients (P:78%, PC:82%) aged between 18 and 34 (P:58%, PC:51%). No statistical difference in mean age was observed between people in prison or people in police custody (P: 34.1, PC:35.6, t=-1.23 (325), *p* = 0.221). Attendances related to OAT were most common for patients in prisons (59%, X^2^ = 14.41, *p* < 0.001), whereas attendances related to heroin were most common for patients in police custody (55%, X^2^ = 6.35, *p* < 0.001). For patients in prison, 90% of attendances were transported to hospital, compared with 70% of attendances for patients in police custody (X^2^ = 12.66, *p* < 0.001). Of all opioid-related attendances (*n* = 328), 64% were harms related to opioid consumption, 35% were harms related to opioid withdrawal, and 6% were related to other and unspecified opioid-related harms (Table 1).

**Table 1:** Patient characteristics of opioid-related ambulance attendances for patients in justice settings in New South Wales, December 2020 – April 2023

Characteristics		Patients in prison (*n* = 78)	Patients in police custody (*n* = 250)	All patients in justice settings (*n* = 328)
Gender	Male	61 (78.2)	204 (81.6)	265 (80.8)
	Female	17 (21.79)	46 (18.4)	63 (19.2)
Age group	18–24	11 (14.1)	20 (8.0)	31 (9.5)
	25–34	34 (43.6)	107 (42.8)	141 (43.0)
	35–44	24 (30.7)	81 (32.4)	105 (32.0)
	45+	9 (11.5)	42 (16.8)	51 (15.6)
Opioid type ^a^	Heroin	30 (38.5)	137 (54.8)	167 (50.9)
	Prescription opioids	*n* < 5^b^	34 (13.6)	*n*≥32 (≥9.8)^b^
	OAT	46 (59.0)	87 (34.8)	133 (40.6)
Nature of attendance ^a,^	Consumption	39 (50.0)	170 (68.0)	206 (63.7)
	Withdrawal	26 (33.3)	88 (35.2)	114 (34.8)
	Other and unspecified harms ^c^	14 (17.9)	5 (2.00)	19 (5.8)
Transported to hospital		70 (89.7)	174 (69.6)	244 (74.4)

^a^ Groups are not mutually exclusive, and a single attendance could involve more than one drug type or nature of attendance

^b^ Values obfuscated to protect patient anonymity

^c “^Other and unspecified harms” includes attendances related to drug concealment, treatment-related incidents, and other unspecified harms

Of all opioid-related attendances for patients in prisons (*n* = 78), 26 (33%) were withdrawal-related harms, 39 (50%) related to opioid consumption and 14 (18%) for other opioid-related harms (Table 2). In 65% of attendances related to opioid withdrawal in prisons, heroin was the primary substance of complaint compared with 47% for patients in police custody. Furthermore, 39% reported withdrawal from OAT (90% from methadone), compared with 51% in police custody (33% from methadone). No patients in prisons reported withdrawal symptoms related to prescription opioids; however, withdrawal from prescription opioids was reported in 9% of attendances for patients in police custody.

**Table 2:** Characteristics of the opioid withdrawal and consumptions attendances for patients in justice settings in New South Wales, December 2020 – April 2023

		Patients in prison	Patients in police custody
Withdrawal (n)		26	88
Gender	Male	16 (61.5)	72 (81.8)
Age (mean, SD)		40 (12.3)	36 (9.2)
Opioid type^a^	Heroin	17 (65.4)	41(46.6)
	OAT	10 (38.5)	45 (51.1)
	Prescription opioids	0 (0.0)	8 (9.1)
Transport to hospital		23 (88.5)	61 (69.3)
Consumption (n)		39	170
Gender	Male	33 (84.6)	137 (80.6)
Age (mean, SD)		31 (7.4)	35 (8.9)
Opioid type^a^	Heroin	8 (20.5)	69 (40.6)
	OAT	28 (71.8)	46 (27.1)
	- Methadone	12 (42.9)	26 (56.5)
	- Buprenorphine^c^	15 (53.6)	20 (43.5)
	Prescription opioids	*n* < 5^b^	34 (20.0)
Drug administration modality	Injected	18 (46.2)	49 (28.8)
	Oral ingestion	15 (38.5)	54 (31.8)
	Snorted/Smoked	7 (17.9)	10 (5.9)
	Unknown	*n* < 5^b^	55 (32.4)
Transport to hospital		35 (89.7)	116 (68.2)

^a^ Groups are not mutually exclusive, and a single attendance could involve more than one drug type

^b^ Values obfuscated to protect patient anonymity

^c^ Please note that the NASS data is unable to accurately distinguish between buprenorphine formulations (sublingual, injectable and naloxone containing) and are therefore presented as one buprenorphine category

Harms related to the consumption of OAT occurred more often in prisons (72%), compared with police custody settings where harms due to heroin (41%) and prescription opioids (20%) were more frequently reported (Table 2). IDU was the most common mode of drug consumption in prisons (46%), followed by ingestion (39%) and snorting/smoking (18%). Ingestion (32%) and unknown mode of consumption (32%) were most commonly reported in police custody settings (Table 2).

### Qualitative results

Three key themes were identified from the qualitative analysis of paramedic case notes: patient trust and mistrust of opioid use in justice settings, systemic barriers to providing OAT and other harm reduction strategies in the justice system, and experiences of withdrawal and care in justice settings. Each of these themes included numerous sub-themes (Table 3).

**Table 3:** Summary of coding framework with example quotes

Theme	Subtheme	Example quotes
1. Trust and mistrust of opioid use in justice settings.	Patients disclose information to paramedics and healthcare workers but not correctional staff	“On arrival, patient sitting in a chair with gaol nurse. According to nurse, patient was observed by his cellmate to stiffen with locked jaw, moaning and groaning, stiff and spaced out and also started dribbling. …When patient does not have the guard nearby patient admits to having Suboxone injection today. Nurse states prisoners keep it quiet from the guards, but Suboxone is in the gaol.”
	Unsafe practices and excess consumption in an attempt to conceal drugs	“Called to male inmate following oral ingestion of 10 × 8 mg buprenorphine strips. Patient states was a deliberate ingestion due to cell search, not a suicide attempt.”
2. Systemic barriers for providing OAT and other harm reduction strategies in the justice system.	Reliance on informal processes (personal networks, friends and family, police capacity) to maintain OAT while in police custody	“Patient last had methadone Monday, now Wednesday morning. Patient concerned that if he does not have methadone he will go through withdrawals. …Reassured patient, advised to call his mother in morning before court to ger her to bring down methadone.”
		“Patient has been in police custody for the 4 days and has not had prescription medication or methadone for the last 3 days. Patient has been taken to emergency department for assessment and referral for methadone script. Patient transported in police vehicle as per police request. Crew followed behind to handover at emergency department.”
		“Called to police station for patient withdrawing for methadone. …Patient states he just needs methadone and there is nothing paramedics can do for him. Police state they will arrange for patient to call someone tomorrow morning to get methadone for him and, if bail refused in court, will be able to get his methadone when taken to corrections facility.”
	Police unable or ill equipped to provide OAT in custody	“Patient was taken into custody this morning and has been showing signs of drug withdrawal. Police had methadone provided to them by the patient but stated they weren’t allowed to let patient take the substance as they can’t confirm it is methadone.”
	New entrants into the justice system particularly vulnerable	“States she [patient] is a daily heroin user and has been in this facility for one day, states on methadone program, patient has not yet received dose – corrections awaiting doctors report to confirm”
		“Patient was denied bail today at the courthouse and is due to go to prison this afternoon. …patient began complaining to the correctional officers that he was having withdrawal symptoms of nausea, as he had not had his Suboxone for 3 days. …Paramedics informed the patient they were unable to provide the patient with Suboxone. Paramedics called [local] hospital who stated they did not have Suboxone as it is a highly restricted drug and would need to go to his regular pharmacy to receive his Suboxone. The correctional officers advised the patient he would likely get his Suboxone in [neighbouring city] when he was seen by Justice Health as they had organised for his script to be sent there.”
		“Patient in a cell in police custody. …Patient states that he has not been able to visit his methadone clinic in the past 48 hours due to being in police custody.”
	People given insufficient dosages as a temporary measure	“Patient states he wants a doctor to review his methadone dose… stating he can’t do it on the dose prescribed…”
	Delays lead to patients using methadone extra-medically	“On arrival, patient in cell has taken some methadone that was his cell mates (not his). Patient says facing longer stint in prison this time so is wanting to sort out some methadone for himself, so he doesn’t resort to other means.”
	Limited harm reduction options for prisoners	“Multiple patients (some hepatitis C positive) had injected methadone that had been spat out after it was given…Syringe rinsed with Fincol between injections.” “Patient admitting to using ‘dirty’ fit pack today to inject buprenorphine…”
3. Experiences of withdrawal in the justice system.	Signs of physical and psychological distress experienced by patients withdrawing from opioids	“Patient has had five days of withdrawal symptoms whilst in facility. …Patient has gone without alcohol/drugs since arriving…staff state patient has increased in agitation, aggression, delirium, and visual and audible hallucinations.”
		“…complaints of abdominal pain, hot cold sensations, headache and pain making it hard to breathe, patient states active withdrawal from suboxone… Suboxone taken one day ago and takes every day and concerned will get worse.”
		“Patient [hepatitis C reported] has been withdrawing… vomiting and abdominal pain for 5 days…today patient has also experience palpitations…staff handover state patient has been vomiting green fluid…”
	Current systems are unable to safely manage overdose and withdrawal symptoms	“Registered nurse states increasing severity of symptoms to be unmanageable by facility, requesting hospital transfer for further medical management of withdrawal symptoms.”
		“Heroin overdose …Staff concerned if [patient] administered naloxone, no medical staff on after 17:00 to monitor patient.”
		“Patient has many years of opiate abuse now having withdrawal issues. Reason for transfer: registered nurse medical staff finish shift at 21:00 with patient needing monitoring overnight…no registered nurse on duty overnight…patient transported for overnight care.”

Multiple incidents requiring paramedic attendance involved unsafe drug use practices and excessive consumption motivated by patients’ fear of prison staff discovering their possession of drugs, with one case even involving the deliberate ingestion of drugs for concealment purposes in response to a cell search. These behaviours included concealing substances and drug paraphernalia and refusing to discuss recent opioid use or personal histories of substance use, compromising the quality and safety of care provided (Table 3). Patients in prisons demonstrated more trust and a greater willingness to disclose information about their opioid use to paramedics and prison nurses while avoiding these discussions in the presence of correctional staff.

Ambulance case notes detailed systemic barriers to providing OAT and other harm reduction strategies in justice settings. Multiple cases highlighted the difficulties in implementing policies and procedures for providing OAT in police custody. Patients instead relied on informal processes, such as personal networks, friends and family, and police capacity and willingness, to maintain OAT while in police custody. New entrants to the justice system, who moved between police custody, the court system, and prison, were particularly vulnerable to disruptions in their treatment. Medical evaluations often took days or weeks, resulting in paramedics and emergency departments having to facilitate medication and dosage reviews, prescriptions, and general medical advice. By this time, patients had often entered states of moderate to severe opioid withdrawal. Patients, healthcare staff, and correctional staff highlighted the lack of timely access to healthcare professionals and services within justice settings to verify patients’ prescribed medications, histories of substance use disorders, or participation in OAT services in the community. One patient with existing community ties to an OAT was described as “*…a daily heroin user and has been in this facility for one day*,* states on methadone program*,* patient has not yet received dose – corrections awaiting doctors report to confirm*”, and another patient is described as being *“in police custody for the last four days and has not had prescription medication or methadone”* highlighting prolonged disruptions to continuity of care in justice-settings. These delays were known among some of the patients with prior justice system involvement, leading some to acquire diverted methadone or buprenorphine to self-medicate and avoid withdrawal. In some cases, the use of diverted OAT was accompanied by risky drug use behaviours. For example, patients described severe side effects after sharing a syringe filled with *“spat out methadone”* with other people in prison, despite knowing that at least one person was hepatitis C positive. During this incident, they described attempting to mitigate these risks by rinsing the syringe with *Fincol*, a hospital disinfectant available in NSW prisons (Table 3).

Patients experienced significant physical and psychological distress due to opioid withdrawal, typically while being denied access to OAT. Many patients had detailed clinical case notes describing their withdrawal symptoms including *“…complaints of abdominal pain*,* hot cold sensations*,* headache and pain making it hard to breathe”* and *“agitation*,* aggression*,* delirium and visual and audible hallucinations*”.Prison health staff were often unable to appropriately respond to withdrawal symptoms and overdoses due to staffing limitations and finite resources, particularly late at night and early in the morning. The severe withdrawal symptoms experienced by patients in justice settings reflect poor continuity of care, stemming from systemic issues, ineffective procedures for maintaining OAT during transitions into the justice system, and the complexities of providing quality care for opioid use disorders in justice settings (Table 3).

## Discussion

This study described patterns of opioid-related harms for people who were either in a prison or in police custody in NSW between December 2020 and April 2023 using ambulance data from the NASS. It demonstrated that harms experienced were attributable to both opioid withdrawal and opioid consumption-related presentations. Taken together, the contextualisation of these findings utilising qualitative methods underscored pressure points in current models of care for individuals transitioning into and out of the justice system who are living with opioid dependence. These results have implications for harm reduction in justice settings, including greater continuity of care in providing more streamlined access and availability to OAT for people in prison, piloting of prison-based needle syringe programs, and new approaches to screening and treatment for drug and alcohol dependence within justice settings [13]. Our study was novel in its use of ambulance data to describe patterns of acute opioid-related harms in NSW justice settings. Previous studies have demonstrated the utility of ambulance attendance data for tracking opioid-related harms in the community [25], however this is the first known study to employ such methods to describe opioid-related harms in people in justice settings.

### Opioid consumption and withdrawal in justice settings

The international justice health literature highlights that people who use opioids or live with an opioid use disorder (OUD) experience higher rates of incarceration and recidivism [29]. In a 2015 survey study conducted in NSW prisons, access to drugs are described as “*quite easy*” or “*very easy*” to acquire in NSW prisons, where approximately 9% and 13% of people in prison report using prescription analgesics and heroin, respectively, while incarcerated [11]. Findings from our study showed that harms related to the consumption of opioids in NSW prisons were reported approximately 50% of the time. When consumption-related harms were reported to paramedics, 72% of these harms in prisons were related to OAT, with a majority reporting extra-medical methadone (43%) or buprenorphine (54%) consumption. This finding was less pronounced in police custody settings with less than 30% of attendances involving consumption of OAT prior to arrest (27%), however a higher proportion involving heroin (41%) was subsequently reported. Diversion or extra-medical use of OAT in NSW prisons has previously been reported by one-third (35%) of respondents to a prison health survey [10, 11]. Our findings align with previous qualitative research that highlights the reasons for diversion and extra-medical use of OAT is a complex issue that requires a nuanced understanding of the social landscape of drug consumption in prisons. This includes non-disclosure and extra-medical use to combat the negative effects of opioid withdrawal by individuals receiving care for fear of criminal sanction [15, 26], and pressures from other people in prison to divert OAT [19, 30].

The introduction of OAT across prisons has largely been beneficial in reducing heroin and extra-medical opioid use, intravenous drug administration, syringe sharing, and the incidence of overdoses [31, 32]. Furthermore, the availability of OAT medications inside prisons have observed benefits in reducing drug-related mortality while in prison [33] and upon release [34– 36]. NSW has the largest number of people receiving OAT in correctional facilities in Australia, with over 3,600 people in prison receiving treatment in 2023 (76% receiving buprenorphine, 20% receiving methadone and 5% receiving long-acting injectable buprenorphine) [18]. Although proportions of attendances related to OAT-consumption in prisons was high in this study (72%), we also identified that approximately one-third (33%) of patients had an ambulance attendance related to opioid withdrawal symptoms. Of those withdrawal attendances 65% were related to heroin and 39% were related to OAT.

Opioid withdrawal is not only a physically painful experience, but also associated with serious psychological distress that is known to increase the risk of death by suicide in prisons during the first week of incarceration [33, 37]. Furthermore, international conventions stipulate that people in prison should not be deprived access to healthcare, and it has been shown that access to OAT in prisons significantly lowers the risk of mortality in those who are opioid-dependent [33, 38, 39]. Navigating the justice-health system, including accessing an OAT program, is especially challenging for individuals who had not previously received OAT in the community [13, 19, 36]. Several clinical accounts from both police custody settings and prisons in our study detail patients requesting access to their OAT or asking when they will be placed on an OAT program – many of whom have already begun experiencing symptoms of opioid withdrawal. In many of these cases, OAT was denied in police custody until designated protocols could be followed and, during prison entry, patients were, at times, waiting weeks to be assessed and placed on an OAT program.

Patients who did not have pre-existing links to an OAT program in the community were particularly vulnerable to extra-medical OAT use: “*patient [in cell] has taken some methadone that was his cell mates (not his). Patient says facing longer stint in prison this time so is wanting to sort out some methadone for himself*,* so he doesn’t resort to other means.”* One Australian qualitative study explained that when people were unable to access OAT while in prison, some turned to *“chasing drugs”* to avoid withdrawal, and for those on an OAT program, felt as if they were easily identifiable and made them targets for pressure to divert their prescribed dose [19]. To combat people in prison turning to illicit drugs to handle opioid withdrawal, there is a need to ensure procedures and adequate resources are in place to identify and assess all people entering the prison system who may be at risk of opioid withdrawals and assess them for OAT. Australian prisons need to consider ways to improve continuity of care for opioid users who are already prescribed OAT in the community, such as the need to ensure adequate clinical and administrative staff availability to enable the timely confirmation of OAT with previous prescribers.

### Consequence of opioid use in justice-settings and the role of emergency services

In our study, drug consumption in prisons largely occurred via IDU (46%) compared with mixed modes of consumption in police custody. Several physical risks including BBV transmission, cellulitis and other infections have been associated with IDU [40, 41] and were also highlighted in the clinical case notes of patients in this study. Examples of physical risks in our study included accounts of syringe sharing, where paramedics described that *“multiple patients (some hepatitis C positive) had injected methadone that had been spat out…”*. The use of Fincol, a disinfectant provided in NSW prisons for the use of syringe disinfecting, highlights an attempt by patients to reduce the risk of BBV transmission associated with sharing injecting equipment. Despite its efficacy for reducing BBV transmission, people in NSW prisons have reported challenges accessing Fincol and finding time to complete the disinfectant process without attracting the attention of prison staff [42, 43]. There is evidence from the community that suggests OAT used in combination with needle and syringe programs reduces risk of hepatitis C transmission, however studies are yet to confirm this in prison settings [20, 44]. Currently, harm reduction strategies including long-acting injectable buprenorphine and hepatitis C testing and treatment programs have been rolled out in Australian prisons, however there are ongoing calls to simplify the testing and treatment pathways for hepatitis C and rapidly scale-up these programs [20]. Furthermore, prison-based needle syringe programs have been piloted in many prisons around the world, with encouraging results in reducing harms associated with opioid use and IDU, and should be piloted in an Australian setting [20, 41, 45].

Covert acquisition of illicit drugs, OAT diversion, and the use of unsterilised injecting equipment are known to occur in prisons [19, 40, 46]. The secrecy surrounding drug use and possession, and possession of drug paraphernalia in prisons was evident in many of our clinical case notes, with many patients reluctant to divulge their use of an opioid or their method of administration in the presence of corrections staff, possibly out of fear of punishment or as a way of preserving their drug supply channel. Once patients were being transported to hospital via ambulance (90%), they were noticeably more forthcoming with paramedics about the substance they took and how it was administered, highlighting the pivotal role paramedics play in justice health.

It was evident in our study that current systems for handling opioid withdrawal and overdose in justice-settings face significant barriers, including after-hours care. Paramedic notes included examples of corroboration by nurses who noted *“staff concerned if [patient] administered naloxone*,* no medical staff on after 17:00 to monitor patient”* and concerns surrounding ongoing care *“requesting a hospital transfer for further medical management”* and detailing that *“no registered nurses [are] on duty overnight…”*. These accounts underscore the effects finite resources have on continuity of healthcare in correctional settings when dealing with drug withdrawal and overdose, and the reliance justice-health services have on ambulances, where 90% of all attendances occurring in prisons in our study required the patient to be transported to hospital, compared to 65% of opioid-related attendances in community settings [25]. Continuity of healthcare in justice settings, including sufficient resourcing of healthcare professionals, AOD and overdose education and training including naloxone provision, and screening and communication of treatment options for OUD and other AOD disorders are essential aspects to delivering good healthcare in justice settings [13, 20, 32, 47]. Moreover, these factors also play an important role in mitigating transmission of BBV while under the care of the justice system, as well as overdose prevention and OAT retention after leaving prison [13, 20, 32, 47].

### Limitations

This study analysed data from NSW ambulance presentations and therefore the results may not be generalised to other jurisdictions both in Australia and internationally. Like other public health surveillance datasets, the NASS uses coded administrative data derived from paramedic clinical acumen and details revealed by the patients or present third parties (e.g., police, prisons guards, prison nurses and other incarcerated people) at the time of attendance and are collected for administrative and clinical purposes rather than for research. As a result, patient care records could be incomplete, inconsistent, or inaccurate due to non-standardised methods of data collection (Lubman, Matthews et al., 2020). Furthermore, there is likely a reluctance for people in prison to disclose the use of opioids or diversion of prescribed substances for extra-medical use. Estimates in this study are conservative, as only those people in prison attended to by an ambulance were included, acknowledging that people in prison can seek and receive medical care in other ways (e.g., prison health clinics and transfers to hospital). This study did not control for the number of patients transitioning through the justice health system during the study period, therefore prevalence could not be determined. Variables related to Indigenous status or ethnicity are not available in the NASS dataset, however we acknowledge the high rates of Aboriginal and Torres Strait Islander incarceration in Australian prisons and their likely overrepresentation in our dataset. Similarly, BBV status is not well collected during an ambulance attendance and paramedic records lack information on infection status, treatment status, temporality of infection and/or reinfection [48].

## Conclusion

Our study demonstrated the utility of administrative ambulance data in describing and contextualising opioid-related harms for people in justice settings in NSW. Our findings highlight the need for targeted harm reduction programs including prison-based needle and syringe programs, and revised policies that work towards guaranteeing safe and consistent access to treatment for opioid dependence and associated harms, such as sufficient resourcing and training of correctional and health care staff, AOD and overdose education and training, and continued naloxone provision, particularly during transitions in and out of justice settings.

## Data Availability

The datasets generated and analysed for this study are not publicly available as a requirement to protect privacy and confidentiality of patients. Ambulance data are provided to Turning Point under strict conditions for storage, retention and use. Researchers who would like to undertake additional analyses of the data are invited to contact Turning Point as the data custodians at aodstats@turningpoint.org.au.

## Abbreviations

NSW: New South Wales
NASS: National Ambulance Surveillance System
OAT: Opioid agonist therapy
AOD: Alcohol and other drugs
BBV: Bloodborne virus(es)
IDU: Injecting drug use
OUD: Opioid use disorder

## References

[1] Biondo S, Young J. Inequalities and inequities experienced by people with mental health and substance use issues involved in the criminal justice system. VAADA & The University of Melbourne; 2019. 2019/07/05/.

[2] Fazel S, Seewald K. Severe mental illness in 33 588 prisoners worldwide: systematic review and meta-regression analysis. Br J Psychiatry. 2012;200(5):364–73. 22550330 10.1192/bjp.bp.111.096370

[3] Fazel S, Ramesh T, Hawton K. Suicide in prisons: an international study of prevalence and contributory factors. Lancet Psychiatry. 2017;4(12):946–52. 29179937 10.1016/S2215-0366(17)30430-3 PMC6066090

[4] Cunningham E, Hajarizadeh B, Bretana N, Amin J, Betz-Stablein B, Dore G, et al. Ongoing incident hepatitis C virus infection among people with a history of injecting drug use in an Australian prison setting, 2005‐2014: the HITS‐p study. J Viral Hepatitis. 2017;24(9):733–41. 10.1111/jvh.12701 28256027

[5] Dolan K, Wirtz AL, Moazen B, Ndeffo-Mbah M, Galvani A, Kinner SA, et al. Global burden of HIV, viral hepatitis, and tuberculosis in prisoners and detainees. Lancet. 2016;388(10049):1089–102. 27427453 10.1016/S0140-6736(16)30466-4

[6] Graham L, Fischbacher CM, Stockton D, Fraser A, Fleming M, Greig K. Understanding extreme mortality among prisoners: a national cohort study in Scotland using data linkage. Eur J Public Health. 2015;25(5):879–85. 25678604 10.1093/eurpub/cku252

[7] Kariminia A, Butler TG, Corben SP, Levy MH, Grant L, Kaldor JM, et al. Extreme cause-specific mortality in a cohort of adult prisoners—1988 to 2002: a data-linkage study. Int J Epidemiol. 2007;36(2):310–6. 17158524 10.1093/ije/dyl225

[8] European Monitoring Centre for Drugs and Drug Addiction. Penalties for drug law offences in Europe at a glance. Lisbon: EMCDDA; 2019. 2019/05/13/.

[9] Enggist S, Møller L, Galea G, Udesen C. Prisons and health: World Health Organization. Regional Office for Europe; 2014.

[10] Justice Health Forensic Mental Health Network. People in NSW Public Prisons: 2020 Health Status and Service Utilisation Report. NSW Health; 2022 2022/11//. Report No.: 1.

[11] Justice Health Forensic Mental Health Network. 2015 Network Patient Health Survey Report. New South Wales, Australia: NSW Health; 2017 2017/05//. Report No.: 1.

[12] O’Keefe D, Ritter A, Stoove M, Hughes C, Dietze P. Harm reduction programs and policy in Australia: barriers and enablers to effective implementation. SUCHT. 2020;66(1):33–43.

[13] Grella CE, Ostile E, Scott CK, Dennis M, Carnavale J. A scoping review of barriers and facilitators to implementation of medications for treatment of opioid use disorder within the Criminal Justice System. Int J Drug Policy. 2020;81:102768. 32446130 10.1016/j.drugpo.2020.102768 PMC8372195

[14] Komalasari R, Wilson S, Haw S. A systematic review of qualitative evidence on barriers to and facilitators of the implementation of opioid agonist treatment (OAT) programmes in prisons. Int J Drug Policy. 2021;87:102978. 33129135 10.1016/j.drugpo.2020.102978

[15] Winetsky D, Fox A, Nijhawan A, Rich JD. Treating opioid use disorder and related infectious diseases in the Criminal Justice System. Infect Dis Clin N Am. 2020;34(3):585–603. 10.1016/j.idc.2020.06.012 PMC7433020 32782103

[16] Agramunt S, Lenton S. Evaluation of the western Australian Police Force Naloxone Pilot: a summary of the main findings. National Drug Research Institute; 2023.

[17] Recent overdose deaths. raise fears for people in prison – naloxone must be made available to save lives [press release]. 2024.

[18] Australian Institute of Health and Welfare. National Opioid Pharmacotherapy Statistics annual data. 2024.

[19] Marshall AD, Schroeder SE, Lafferty L, Drysdale K, Baldry E, Stoové M, et al. Perceived access to opioid agonist treatment in prison among people with a history of injection drug use: a qualitative study. J Subst Use Addict Treat. 2023;150:209066. 37156422 10.1016/j.josat.2023.209066

[20] Winter RJ, Sheehan Y, Papaluca T, Macdonald GA, Rowland J, Colman A, et al. Consensus recommendations on the management of hepatitis C in Australia’s prisons. Med J Aust. 2023;218(5):231–7. 36871200 10.5694/mja2.51854

[21] Fuh L, Hays H, Brown N, Shirk MB. Characterization of drug overdoses in an Ohio incarcerated population. Clin Toxicol. 2016;54(3):266–70. 10.3109/15563650.2015.1135338 26795588

[22] McKendy L, Biro SM, Miron M, Keown LA. Understanding overdose incidents in Canadian federal custody. Int J Drug Policy. 2021;92:103131. 33558166 10.1016/j.drugpo.2021.103131

[23] Lubman DI, Matthews S, Heilbronn C, Killian JJ, Ogeil RP, Lloyd B, et al. The National Ambulance Surveillance System: a novel method for monitoring acute alcohol, illicit and pharmaceutical drug related-harms using coded Australian ambulance clinical records. PLoS ONE. 2020;15(1):e0228316. 32004349 10.1371/journal.pone.0228316 PMC6994147

[24] Ogeil RP, Scott D, Faulkner A, Wilson J, Beard N, Smith K et al. Changes in alcohol intoxication-related ambulance attendances during COVID-19: how have government announcements and policies affected ambulance call outs? Lancet Reg Health – Western Pac. 2021;14. 10.1016/j.lanwpc.2021.100222 PMC8443417 34545354

[25] McGrath M, Stare M, Chua P, Ogeil R, Nehme Z, Scott D, et al. Opioid-related ambulance attendances during the first 2 years of the COVID‐19 pandemic in Victoria, Australia. Addiction. 2024;119(2):348–55. 37816493 10.1111/add.16360

[26] Mitchell SG, Kelly SM, Brown BS, Reisinger HS, Peterson JA, Ruhf A, et al. Incarceration and opioid withdrawal: the experiences of Methadone patients and out-of-treatment heroin users. J Psychoactive Drugs. 2009;41(2):145–52. 19705676 10.1080/02791072.2009.10399907 PMC2838492

[27] Lubman DI, Heilbronn C, Ogeil RP, Killian JJ, Matthews S, Smith K, et al. National Ambulance Surveillance System: a novel method using coded Australian ambulance clinical records to monitor self-harm and mental health-related morbidity. PLoS ONE. 2020;15(7):e0236344. 32735559 10.1371/journal.pone.0236344 PMC7394421

[28] Braun V, Clarke V. Using thematic analysis in psychology. Qualitative Res Psychol. 2006;3(2):77–101.

[29] Winkelman TNA, Chang VW, Binswanger IA, Health. Polysubstance Use, and criminal justice involvement among adults with varying levels of opioid use. JAMA Netw Open. 2018;1(3):e180558. 30646016 10.1001/jamanetworkopen.2018.0558 PMC6324297

[30] White N, Ali R, Larance B, Zador D, Mattick RP, Degenhardt L. The extramedical use and diversion of opioid substitution medications and other medications in prison settings in Australia following the introduction of buprenorphine-naloxone film: use and abuse of medications in prisons. Drug Alcohol Rev. 2016;35(1):76–82. 26331899 10.1111/dar.12317

[31] Hedrich D, Alves P, Farrell M, Stöver H, Møller L, Mayet S. The effectiveness of opioid maintenance treatment in prison settings: a systematic review: opioid maintenance in prison. Addiction. 2012;107(3):501–17. 21955033 10.1111/j.1360-0443.2011.03676.x

[32] Mazzilli S, Royuela L, Tavoschi L, Vandam L, Montanari L. Prevalence of injected drug use and access to OAT in prison: survey in 7 EU countries, 2014–2018. Eur J Pub Health. 2022;32(Supplement3):ckac131416.

[33] Larney S, Gisev N, Farrell M, Dobbins T, Burns L, Gibson A, et al. Opioid substitution therapy as a strategy to reduce deaths in prison: retrospective cohort study. BMJ Open. 2014;4(4):e004666. 24694626 10.1136/bmjopen-2013-004666 PMC3987723

[34] Curtis M, Wilkinson AL, Dietze P, Stewart AC, Kinner SA, Cossar RD, et al. Prospective study of retention in opioid agonist treatment and contact with emergency healthcare following release from prisons in Victoria, Australia. Emerg Med J. 2023;40(5):347–54. 36759173 10.1136/emermed-2022-212755 PMC10176422

[35] Marsden J, Stillwell G, Jones H, Cooper A, Eastwood B, Farrell M, et al. Does exposure to opioid substitution treatment in prison reduce the risk of death after release? A national prospective observational study in England: prison-based OST and mortality after release. Addiction. 2017;112(8):1408–18. 28160345 10.1111/add.13779

[36] Russell C, Pang M, Nafeh F, Farrell Macdonald S, Derkzen D, Rehm J, et al. Barriers and facilitators to opioid agonist treatment (OAT) engagement among individuals released from federal incarceration into the community in Ontario, Canada. Int J Qualitative Stud Health Well-being. 2022;17(1):2094111. 10.1080/17482631.2022.2094111 PMC9258049 35787743

[37] Humber N, Piper M, Appleby L, Shaw J. Characteristics of and trends in subgroups of prisoner suicides in England and Wales. Psychol Med. 2011;41(11):2275–85. 21557891 10.1017/S0033291711000705

[38] Stöver H, Jamin D, Michels II, Knorr B, Keppler K, Deimel D. Opioid substitution therapy for people living in German prisons—inequality compared with civic sector. Harm Reduct J. 2019;16:1–9. 31864356 10.1186/s12954-019-0340-4 PMC6925451

[39] International Covenant on Economic. Social and Cultural Rights, (1966).

[40] Small W, Kain S, Laliberte N, Schechter MT, O’Shaughnessy MV, Spittal PM. Incarceration, addiction and harm reduction: inmates experience injecting drugs in prison. Subst Use Misuse. 2005;40(6):831–43. 15974143 10.1081/ja-200030795

[41] Lazarus JV, Safreed-Harmon K, Hetherington KL, Bromberg DJ, Ocampo D, Graf N, et al. Health Outcomes for Clients of Needle and syringe programs in prisons. Epidemiol Rev. 2018;40(1):96–104. 29659780 10.1093/epirev/mxx019

[42] Treloar C, McCredie L, Lloyd AR. On behalf of the H-pi. Acquiring hepatitis C in prison: the social organisation of injecting risk. Harm Reduct J. 2015;12(1):10. 25903401 10.1186/s12954-015-0045-2 PMC4413553

[43] Hajarizadeh B, Carson JM, Byrne M, Grebely J, Cunningham E, Amin J, et al. Incidence of hepatitis C virus infection in the prison setting: the SToP-C study. J Viral Hepatitis. 2024;31(1):21–34. 10.1111/jvh.13895 PMC10952254 37936544

[44] Platt L, Minozzi S, Reed J, Vickerman P, Hagan H, French C, et al. Needle and syringe programmes and opioid substitution therapy for preventing HCV transmission among people who inject drugs: findings from a Cochrane Review and meta-analysis. Addiction. 2018;113(3):545–63. 28891267 10.1111/add.14012 PMC5836947

[45] Kinder S. Ethic justice: needle and syringe programs in Australian prisons. Med J Aust. 2020(16).

[46] Dolan KA, Wodak AD, Hall WD. Methadone maintenance treatment reduces heroin injection in New South Wales prisons. Drug Alcohol Rev. 1998;17(2):153–8. 16203480 10.1080/09595239800186951

[47] Curtis M, Berk J, Larney S, Rich JD, Stoové M. Switching of opioid agonist treatment modality during imprisonment: a novel marker for increased support need during and following release from prison. Int J Drug Policy. 2022;100:103572. 34998045 10.1016/j.drugpo.2021.103572 PMC8810681

[48] Beard N, McGrath M, Scott D, Nehme Z, Lubman DI, Ogeil RP. Using ambulance surveillance data to characterise blood-borne viral infection histories among patients presenting with acute alcohol and other drug‐related harms. Emerg Med Australasia. 2024 Aug;36(4):536–542. 10.1111/1742-6723.14394. Epub 2024 Feb 28 10.1111/1742-6723.14394 38414361

